# Complementarity As Generative Principle: A Thought Pattern for Aesthetic Appreciations and Cognitive Appraisals in General

**DOI:** 10.3389/fpsyg.2017.00727

**Published:** 2017-05-09

**Authors:** Yan Bao, Alexandra von Stosch, Mona Park, Ernst Pöppel

**Affiliations:** ^1^School of Psychological and Cognitive Sciences and Beijing Key Laboratory of Behavior and Mental Health, Peking UniversityBeijing, China; ^2^Human Science Center, Institute of Medical Psychology, Ludwig Maximilians University of MunichMunich, Germany; ^3^Parmenides Center for Art and SciencePullach, Germany; ^4^Department “Diversity of Forms of Knowledge”, Humboldt University of BerlinBerlin, Germany; ^5^Department of Comparative Cultural Studies, Academy of Music Hanns EislerBerlin, Germany

**Keywords:** complementarity, visual art, neuroaesthetics, brain imaging, taxonomy of functions, modeling, thought pattern, language bias

## Abstract

In experimental aesthetics the relationship between the arts and cognitive neuroscience has gained particular interest in recent years. But has cognitive neuroscience indeed something to offer when studying the arts? Here we present a theoretical frame within which the concept of complementarity as a generative or creative principle is proposed; neurocognitive processes are characterized by the duality of complementary activities like bottom-up and top-down control, or logistical functions like temporal control and content functions like perceptions in the neural machinery. On that basis a thought pattern is suggested for aesthetic appreciations and cognitive appraisals in general. This thought pattern is deeply rooted in the history of philosophy and art theory since antiquity; and complementarity also characterizes neural operations as basis for cognitive processes. We then discuss some challenges one is confronted with in experimental aesthetics; in our opinion, one serious problem is the lack of a taxonomy of functions in psychology and neuroscience which is generally accepted. This deficit makes it next to impossible to develop acceptable models which are similar to what has to be modeled. Another problem is the severe language bias in this field of research as knowledge gained in many languages over the ages remains inaccessible to most scientists. Thus, an inspection of research results or theoretical concepts is necessarily too narrow. In spite of these limitations we provide a selective summary of some results and viewpoints with a focus on visual art and its appreciation. It is described how questions of art and aesthetic appreciations using behavioral methods and in particular brain-imaging techniques are analyzed and evaluated focusing on such issues like the representation of artwork or affective experiences. Finally, we emphasize complementarity as a generative principle on a practical level when artists and scientists work directly together which can lead to new insights and broader perspectives on both sides.

## Complementarity As Generative Principle Within a Historical Frame

“Nobody can clap with one hand only” is an old Chinese saying, and these few words describing complementarity as a generative principle express in a simple metaphor what we would like to suggest as a thought pattern for aesthetic appreciations and for cognitive appraisals or evaluations in general. This knowledge reflecting common sense but sometimes remaining on an implicit level can be extracted from many writings since ages in different cultures. “Without an overview on details, there is no beauty, this daughter of the whole and relation”. This is the free translation of a sentence from “Vorschule der Ästhetik” (Pre-School of Aesthetics) which some 200 years ago was published by Richter (1813) in a well received book at that time. This concept of a necessary complementarity with respect to beauty and aesthetic appreciation has (to the best of our knowledge) never been discussed explicitly within a scientific context, but it can be extracted as an underlying thought pattern if one goes through the history of philosophy and reasoning about the arts in different cultures. It would be a misunderstanding, however, if one argues that “harmony” or “good gestalt” has already been suggested as an equivalent conceptual frame within this context (e.g., Eysenck, 1942; Kintsch, 2012); logically speaking, harmony or a good gestalt may be the consequence of complementarity as a generative principle, not the cause.

Apparently, Fechner (1860), the founder of psychophysics, has copied the title “Vorschule der Ästhetik” (1876) from Jean Paul (as he is known as a writer), but the concept of complementarity as a creative principle was not taken up by him. Quite the contrary, Fechner stressed the asymmetry between the whole (or the gestalt) and the details giving rise to the whole. As he writes (in free translation, page 46): “An aesthetics which does not want to keep itself at a low level, focuses on the whole, and considers the details only be themselves…. The whole goes beyond the details”. As Fechner is considered to be not only the founder of psychophysics, but also of “empirical aesthetics” (which for some researchers is strongly associated with “neuroaesthetics”), it is worth noting that a fundamental concept in this field of scientific endeavor has not received the necessary attention; in fact, it is even conceivable that the monocausal reasoning which is typical for research in psychophysics has prevented the broader view toward aesthetic appreciations. To prevent any misunderstandings it should be noted that we refer to “complementarity as a creative or generative principle”, and not to a “descriptive principle” as it is done in theoretical physics, in particular in quantum mechanics with respect to the wave/particle dualism (Pöppel, 2006).

Complementarity as a creative or generative principle with respect to the evaluation of beauty or human reasoning in general as reflected in moral judgments is indeed nothing new. In the Daodejing ascribed to Laozi (2009) one reads in the beginning of the second chapter: “Everyone knows what beauty is, because of the existence of ugliness. Everyone knows what goodness is, because of the existence of badness”. Thus, what is beautiful and what is good can only be understood because of ugliness and badness. This principle of complementarity to understand beauty corresponds conceptually in the traditional Chinese thinking to the Yin-Yang principle which has maintained its importance as a thought pattern since several thousands years.

The principle of complementarity as creative principle is also a basic feature in the Western tradition; as the German philosopher Cassirer (1923/2008) has shown for the dialogs of Plato (1961), both “eidos” and “eidolon”, the idea and the image have to come together in the arts to create consistency or harmony (in German “Stimmigkeit”; it might be mentioned in passing that a lot of philosophical and scientific literature remains mute for the only English speaking community and, thus, appears to be lost nowadays in scientific discourses). At a later occasion, Cassirer (1942/2011) has analyzed the principle of complementarity (without using this word) as a thought pattern in Plato’s reasoning (in Theaitetos) when he refers for instance to “thinking as a conversation of the mind with itself”. It has to be added that in Plato’s Symposion (211d) beauty as a concept is related to love, indeed sexual desire, and life is only worthwhile to live when we see what is beautiful, and what is indeed meant is a beautiful body. In the Greek tradition, Plato was not even the first to stress complementarity as a principle; it was Heraclitus who said that we discover health because of disease, satisfaction because of hunger; to be awake goes together with sleeping, to be old with young, to be good with bad (as Laozi said), or to be male with female. Unity is created by opposing elements in their relations.

The relationship between thinking and speaking as formulated already by Plato in his remark has remained a conceptual challenge until modern times, and it has also been an issue in literature. The German poet Heinrich von Kleist (1805/1806) wrote some 200 years ago in an essay “on the gradual creation of thoughts while talking” that talking to another person is a way to clarify or even create one’s thoughts. The other person does not have to be a specialist at all, and should not even comment on what one is saying. The mere physical presence of another human being is sufficient to trigger the thought processes and may lead to a conclusion one could not reach alone. This would be indeed a prime example of complementarity as a creative principle. What the poet von Kleist suggested is very similar to a concept developed by Vygotsky (1934/1987). His idea is that thinking and speech are not completely separated, but they are also not identical. Vygotsky maintains that speech does not express thinking, but thinking develops in speaking, and thoughts may change while speaking. It is further claimed in his theory that inner speech developed from external speech via a gradual process of “internalization”.

The relationship between language and thought in modern times is also a central issue by Perlovsky with his many publications and rich ideas (e.g., Perlovsky, 2014, 2015, 2016). In a dual hierarchy model explaining the “physics of the mind” which is meant to establish a new area of science, the link between cognition and language is described. His theoretical reasoning is based for instance on observations of emotional effects and aesthetic appreciations, in particular in music. Important references are made to evolutionary principles like the “knowledge instinct”, and his theoretical concepts are formulated also in a mathematical language. Here are some questions Perlovsky (2016) is asking, and also some answers are given: “What is the difference between cognition and language? How does cognition interact with language? Do we think with words, or only use words as labels when a chunk of a thinking process is complete? The science needs to understand the mechanisms of language and cognition interactions; why are they so independent, and so separate? *Dualmodel* is a fundamental principle of the mind modeling interaction between language and cognition. According to the dual model, every mental model has a cognitive and language parts”. We would like to comment that “language” in this model is treated in a very abstract way not taking into account the different language competences like the word lexica for content and function words, furthermore the syntactic, semantic, phonetic, prosodic, temporal and in particular also the pragmatic competence, i.e., employing speech in a way that takes into account specific situations (Pöppel, 2006). For a encompassing theory linking speaking and thinking (rather than language and cognition) it appears to be reasonable to model the link in particular on this detailed level.

The question of a functional link between thinking and language is also relevant with respect to the differentiation between explicit and implicit knowledge. Perhaps the most famous definition of time comes from Augustinus et al. (397/8/1993) in the eleventh book of his confessions where he writes: “Quid enim est tempus? Si nemo ex me quaerat, scio. Si quaerenti explicare velim, nescio” (What then is time? When nobody asks me I know it. If I have to explain it to somebody who has asked, I do not know). This is a prime example of complementarity as a thought pattern because explicit and implicit knowledge come together (Pöppel and Bao, 2011). This thought pattern happens to apply for many theoretical concepts in psychology which are difficult or impossible to define exactly. If one asks “what is” consciousness or attention, one also employs implicitly a complementary thought pattern. And the same pattern applies to “beauty”. The difficulty to define should not be, however, an excuse not to try the best to reach clarity about what is meant on an explicit level following the first rule of thinking as described by Descartes (1637/1990) in his “Discours de la Méthode”.

If in these different cultures, in the ancient Asian and the ancient Western cultures (which presumably did not influence each other), complementarity as a thought pattern was well established, it can be concluded that it reflects a basic human trait in dealing successfully with the physical and the social world. It shall not come as a surprise then that also in the Arabic and Islamic culture the complementarity principle has played an important role. One of the leading philosophers in this culture has been Al-Farabi (who passed away in 950, and who was characterized as the “second philosopher” after Aristotle). Al-Farabi (2005) analyzed in detail for instance the relationship between elements in thinking and their verbal expressions to either create beauty or ugliness in a speech (p.51). Al-Farabi was probably also the first in human history to create in his book “De Scientiis” a taxonomy of functions which deplorably still does not exist (Pöppel and Ruhnau, 2011).

A prime example for complementarity as a generative principle, and on that basis providing a fundamental thought pattern comes from the philosopher Immanuel Kant (1781/1787) in his “Critique of Pure Reason” with his famous statement: “Gedanken ohne Inhalt sind leer, Anschauungen ohne Begriffe sind blind. Daher ist es ebenso notwendig, seine Begriffe sinnlich zu machen, als seine Anschauungen verständlich zu machen”. (Our translation: Thoughts without content are empty, perceptions without notions are blind. Therefore it is equally necessary to make one’s notions sensory as one’s perceptions understandable).

Thus, the thought pattern of complementarity in different cultures exists since ages, and this thought pattern has also been related to aesthetic appreciations. Surprisingly, this principle has not arrived in modern times, or it has been overlooked in its relevance, in spite of the important work of Worringer (1908/2007) on abstraction and empathy, or the thinking of Kandinsky (1912/2009) when he talks in his book “On the Spiritual in Art” (originally written in Russian and German) about creating harmony in pieces of art; in fact, the entire book of Kandinsky can be understood as an expression of complementarity as a creative principle. One might argue that such ideas are historically too far away and not being relevant anymore. Or one might argue that employing “Occam’s razor” to look for simplest explanations, complementarity as a thought pattern is just too complicated. Both arguments (and possibly others) are not really satisfying. One can extract the thought pattern of complementarity as a creative principle in philosophy and in art science also for more recent times or for modern art in general, and many examples have been provided by Liessmann (1999). As a perhaps not so recent example may serve the Spanish philosopher Ortega y Gasset (1916/1957) who in an essay analyzes the beauty of a lady who sits with him in a street car. As he says within 3 s only, (which has been found to be an important time window in cognitive processing: Pöppel, 2009a; Pöppel and Bao, 2014; Wang et al., 2016) Ortega “knows” that the lady is beautiful. In this first impression he is relying on implicit or tacit knowledge (Pöppel and Bao, 2011). The point Ortega makes is that although separate features of the lady’s face are not beautiful or may not be beautiful, it is the relation between the elements that create a gestalt, and which together in their constellation create the beauty of the lady. This analysis is itself an example of complementarity as the first impression based on implicit knowledge is related to an analyzing process representing explicit knowledge. Both knowledge systems interact and have to come together, and as has been suggested previously (Pöppel and Bao, 2011), they are unified both by the aesthetic and the mimetic principle.

## Complementarity As Generative Principle in Neural Processing

The fact that complementarity has not been taken up in empirical aesthetics is particularly surprising as basic features of cognitive processing reflect this principle; many examples by Kelso and Engstrom (2006), and so-called models (see below) refer to such processing. It is a truism that neural information processing to create for instance visual representations is both bottom-up and top-down. It is of course a challenge in research to analyze the different processing aspects, but perception in any modality cannot be understood only within the frame of bottom-up *and* of top-down (e.g., Zhou et al., 2016). Another example in cognitive processing is the complementarity of content and logistical functions (Pöppel, 1989a); as the words indicate, content refers to “what” is represented in percepts, memories, emotions, or volitions, and logistical or “how”-functions refer to the activation of neural systems as reflected in circadian rhythms, attentional control with its different neural implementations (e.g., Bao and Pöppel, 2007), or temporal processing with its different time windows (e.g., Bao et al., 2015). Another example of complementarity refers to anthropological universals and cultural specifics (Bao and Pöppel, 2012); humans (and other higher level organisms) enter the world with genetic programs of possibilities, and during early phases of life specific programs are selected and imprinted, and others are switched off on the basis of the environmental conditions in the natural and cultural sphere. Thus, our value systems and what might be appreciated as beautiful is necessarily both, genetically determined and culturally imprinted. Another important example for complementarity as generative principle is the reafference principle (von Holst and Mittelstaedt, 1950); for behavioral control an efference copy of a motor action has to be balanced by the reafference after execution of the action; only then does the organism obtain a signal that an action has been completed successfully. This mechanism also shows that activities are necessarily future oriented as the potential success of an action is anticipated. Reward of behavior (Arias-Carrión and Pöppel, 2007; Arias-Carrión et al., 2010) is embedded in such reafference systems and is characteristic for any kind of behavioral control, thus, including aesthetic appreciations.

Many more examples could be given, but only one more shall be mentioned which is relevant in particular for aesthetic appreciations, i.e., the complementarity of discrete and continuous time. It has been shown with a number of different experimental paradigms that temporal perception is discrete on a presemantic level of processing (Pöppel, 2009a; Zhou et al., 2012; Bao et al., 2016b). The brain creates discrete time windows on different levels of processing, one being observed for instance in the domain of approximately 3 s. This time window has been proven to be important for poetry (Turner and Pöppel, 1988) or for music (Pöppel, 1988, 1989b); both verses and musical motives are preferably represented by artists in a time window of approximately 3 s, (which should be considered as an operating range and not a physical constant), and violations of this temporal structure by speeding up or slowing down too much verbal or musical representations result in negative aesthetic appreciations. The time window of 3 s which is pre-semantically provided by neural processes, and the expression of a musical motive or the verse in a poem is, thus, also an example of complementarity. Of course, other factors play also an important role in the aesthetic appreciation of music and poetry (e.g., Koelsch, 2014; Jacobs, 2015; Willems and Jacobs, 2016). Although temporal processing is discrete on a basic processing level, we still have the impression of continuity of time. This feeling of continuity turns out to be an illusion, although a necessary illusion (Pöppel and Bao, 2014); it is based on the semantic connection of what is represented in segmented time windows which follow each other, and it serves the purpose (in our view) to create and maintain long-term identity for percepts, concepts and even the self (Pöppel, 2010; Zaytseva et al., 2014; Zhou et al., 2014).

When referring to the “self” we would like to submit the hypothesis that the maintenance of personal identity across time using different time windows (Bao et al., 2015) is based on a specific complementary mechanism which involves the phenomenon of the doppelgänger (Pöppel, 2006). When analyzing one’s own episodic memory almost everybody reports that he or she is pictorially present in the images of the past. This is of course physically impossible; if one sees something one is not represented in the picture. Thus, images in episodic memory do not copy veridically what has been visually experienced; the image of oneself is projected at a later stage into episodic memory, presumably for good psychological reasons. As a consequence when one makes a time travel to the past one also meets one’s own doppelgänger. The complementary mechanism with respect to creation and maintenance of identity suggested is, thus, that “I” am “I” because I am also my own doppelgänger. The tragedy of a memory loss in certain forms of dementia is also the loss of being oneself because the doppelgänger is no longer available. It can be argued that a derivative of this complementary principle can be seen in the desire to obtain self-portraits or “selfies” (Carbon, 2017). To own an external picture of oneself is not necessarily an expression of overdone narcissism but an external means to confirm of one’s own identity mimicking the doppelgänger phenomenon.

Although there is strong evidence for the thought pattern of complementarity within the historical context and as an operating feature in neural processing, this concept has to the best of our knowledge not been acknowledged appropriately for aesthetic appreciations or in cognition in general. In fact, a complementary view toward the behavioral and neural level is sometimes even rejected. Palmer et al. (2013) argue that with respect to “visual aesthetics and human preference” the behavioral level comes first, and analyses of “beauty” on the neural level have to use the behavioral level as an orientation. This is not quite satisfying as the authors also state that clear definitions of beauty are not possible (see above) which makes it certainly not easy to orient oneself. The difficulty to define “beauty” (in German “Schönheit”) has already been spelled out by Fechner (1876): “A simple indicator what makes things beautiful in a broad or narrow sense does not exist; however, there are many attempts, to describe the essence or the kernel of beauty from this or that perspective by a simple phrase” (translation by the authors; p. 10). But whatever “beauty” may be, according to Fechner it is pleasure oriented, not too dissimilar from the view of Plato.

## Different Paradigms Instead of a Taxonomy of Functions: Consequences for Modeling

The greatest problem for empirical aesthetics, and for psychology and brain science in general, is the lack of a taxonomy or classification of functions. It may sound strange, if one says about the own field of research that it misses the most important feature of a science, but this is unfortunately true: We do not have a taxonomy of those functions which we study. Biology or chemistry became accepted sciences after such taxonomies had been developed, but psychology does not have a generally accepted classificatory system. Even worse: Most researchers are not even aware of this fact. Instead of a taxonomy, we are dealing with different conceptual frames or paradigms (Kuhn, 1962; Pöppel and Ruhnau, 2011) which dominate implicitly the reasoning. We would like to remind ourselves of some such paradigms.

The classical paradigm is oriented toward physics, and psychophysics as it was developed some 150 years ago (Fechner, 1860) is still present and quite dominant in the rational conjectures about an understanding of cognitive mechanisms (Stevens, 1975). From a psychological point of view this conceptual frame is based on a physical description of the world, and the task of the researcher is to map the physical world onto the world of subjective representations by defining objective expressions of such relationships. Thus, one is dealing with a physical and not with a psychological “taxonomy”. Another conceptual frame is based on the assumption that cognitive phenomena are directly mapped onto language. This paradigm is deeply rooted in Western culture, mainly based on the thinking of Descartes (1637/1990). Within this concept it is believed that we can navigate through life only with explicit knowledge, and this knowledge is veridically represented in language. That the linguistic paradigm does not include the entirety of what makes humans human, can be derived from an analysis of the different knowledge systems (Pöppel and Bao, 2011). In addition to explicit knowledge our mental apparatus is characterized by implicit knowledge, which is fundamental for aesthetic appreciations; and if one wants to follow Zeki (1999) in his argument (which we do) one has to refer to sensory and visual representations in particular as a third knowledge system. The linguistic paradigm represents only a partial set of what goes on in the human mind and can, thus, not be taken as a taxonomy of functions. A derivative of the linguistic paradigm is a common sense paradigm based on the unreflected use of language, and one can also refer in this case to a “textbook paradigm”. Humans have apparently a natural tendency to ontologize their mental environment by the invention and uncritical use of words. Looking at textbooks, the titles of the chapters imply a classification of functions. One implication of these different entries is that they represent independent mental phenomena like sensation, perception, memory, emotion, attention, motivation, action, intelligence, decision, or consciousness; all these terms show up then in models developed for aesthetic appreciations. Fact is that this textbook paradigm actually dominates the scientific activities in psychology and neuroscience. We would like to indicate only one overlooked problem of this unreflected paradigm, i.e., the conceptual mistake to treat perception and attention on the same categorical level; perception belongs to content function, while attention belongs to the logistical function which provides an operating basis for the creation of content; it is in our view necessary to logically distinguish between content (the “what”) and logistical functions (the “how”) in neural systems (Pöppel, 1989a; Bao and Pöppel, 2012).

And there are more such paradigms which have value within their own frames of reference, and they have been useful to gain some insight into psychological processes, like the psychoanalytical paradigm (Freud, 1932/1961) or the ethological paradigm (Lorenz, 1943), but again only a partial set of the mental machinery is reflected within these paradigms, although in both cases questions of aesthetics have been reflected. Finally, one can mention the neuropsychological paradigm which indeed is favored by ourselves. This paradigm refers to the unique opportunity provided by observations with neurological or psychiatric patients (e.g., Milner and Teuber, 1968; Luria, 1973). The loss of functions in defined patient groups allows the creation of a catalog of functions, and on that basis a reliable classification or taxonomy of functions has already been developed in a rudimentary form. The reasoning in this paradigm is that the loss of a function proves its existence; only what exists can also be lost. Obviously, from an epistemological point of view this paradigm reflects a pragmatic monism in contrast to the classical dualism (e.g., Descartes, 1637/1990) which in a disguised way can still be detected in psychology and neuroscience, as well as in art science.

What are the consequences for empirical aesthetics or neuroaesthetics that we do not own a taxonomy of functions? The answer depends on the perspective one is taking. With a pragmatic attitude one can argue that one does not care about this conceptual deficit, and that one follows a line of research within a preferred paradigm, be it on an explicit or implicit level. Problems arise, however, if one develops models which are meant to represent cognitive processes, in particular with respect to aesthetic appreciations.

This raises the question of typical characteristics for models which happens to be a controversial issue in spite of the important analysis of models for instance by Jacobs and Grainger (1994) within the special area of visual word recognition. We would like to argue that models should be simple, exact, similar (to what is modeled), and fruitful (allowing predictions). If the criteria of being simple and exact cannot be fulfilled, the least one has to demand is that models have to be similar to what is modeled as has been pointed out for instance by Holland (2017) when he refers to exploratory models in comparison to data-driven models (as for weather prediction) and existence-proof models which have also to be exact. However, if one looks at some models as they are promoted in empirical aesthetics (e.g., Leder et al., 2004, 2015; Leder and Nadal, 2014; Redies, 2015), they do not match the necessary criterium of similarity. Suppes (1960, p. 13) has been rather outspoken and may be overdoing the case when he writes: “The attempt to characterize exactly models of an empirical theory almost inevitably yields a more precise and clearer understanding of the exact character of the theory. The emptiness and shallowness of many classical theories in the social sciences is well brought out by the attempt to formulate in any exact fashion what constitutes a model of the theory. The kind of theory which mainly consists of insightful remarks and heuristic slogans will not be amenable to this treatment. The effort to make it exact will at the same time reveal the weakness of the theory”.

As there is no taxonomy of functions in psychology, the models proposed violate the principle of similarity because it is unclear to what they could be similar. The models proposed are certainly not exact or simple. They refer to the repertoire of all psychological functions one can imagine, and they are also not specific to the arts or to aesthetic appreciations as one can replace these terms by others like “fashion” or “design”. A particular deficit is seen in temporal processing (Leder and Nadal, 2014) as it remains unclear how different and sequential mental states can follow each other; how is the end of one mental process defined such that the next one can take over? One is left with the impression of a hidden epistemological dualism. We are well aware of our severe criticism, but in our view this problematic issue has to be raised. What is proposed as “models” is much too general, and it does not match the criteria which define scientific models.

This critical position that one cannot talk about models in neuroaesthetics is based also on another shortcoming; when one attributes any psychological function to specific brain areas or neural networks using fMRI, it is not known whether the BOLD signals reflect excitation or inhibition. On the cellular and intercellular level excitatory and inhibitory transmitters determine neural processing. It is hard to believe that this basic principle of information processing is lost on a modular level, i.e., within circumscribed regions of the brain which are made visible in their activities with imaging technologies. One can only say that some areas participate (more or less) in certain tasks, but the polarity is not known. Changes in activations can mean very different things which are open to any kind of interpretation. They may represent neural excitation within a network, but it could also be inhibition; however, the neural participation could also mean central fatigue, or alternatively increased effort to deal with the complexity of neural processes. All these problems make it difficult if not impossible at present to develop neural models of aesthetic appreciations.

## Some Results on Research in Visual Art and Cognitive Neuroscience

### Neuroscience and Psychology Approaching Art

Given the problematic situation of empirical aesthetics as outlined above we adopt, however, a pragmatic attitude and summarize some research results having in mind that they are rather preliminary with respect to complementarity as a thought pattern, and also with respect to the lack of a taxonomy of functions. It is hoped that in the future these observations and many others that have been obtained may contribute to the development of a taxonomy of functions, and that they can be reflected within the concept of complementarity as a creative or generative principle. The intersection of neuroscience and art has been termed “neuroaesthetics” in recent years, the broader perspective being empirical or experimental aesthetics. While the term neuroaesthetics is rather recent, the scientific approaches are not that new. For instance, in the early 1980s, the Werner-Reimers-Foundation in Bad Homburg (Germany) established a study group to investigate the “Biological Aspects of Aesthetics” in a cross-disciplinary way. Results of this study group have been published in “Beauty and the Brain” documenting the power of interdisciplinary work (Rentschler et al., 1988) with a strong bias toward the aesthetic theory of Kant (1790).

According to Kandel and Mack (2003) there are two general perspectives as to how neurosciences can approach the arts. The first way is to investigate the “cognitive and perceptual analysis of viewing a picture”, the second focuses on “the biology of judgment, taste, aesthetic sensibility”. Thus, the first is interested in the processes that occur in the brain when we look at a work of art, and the second is interested in the (affective and cognitive) responses in the viewer, i.e., in what it is that makes the viewing of artworks a special perceptual experience. It remains, however, rather enigmatic why these two different perspectives should be treated independently; the point in this field of endeavor is to relate the neural with the experiential domain, and harvest in a bidirectional way insights as indicated for instance in the neuropsychological paradigm (see above).

In a review, Chatterjee (2011a) gives a critical overview on the nature of neuroaesthetic activities, and he sets an agenda for further investigations to shift the way how questions are asked and for continuous attempts at refining research methodology and tools. Similarly to Kandel and Mack (2003), Chatterjee proposes a differentiation between two branches of how the neuroscience approaches the arts, i.e., parallelism and experimental neuroaesthetics. While Kandel and Mack differentiate the fields with regards to the questions that are asked, Chatterjee’s distinction is mainly concerned with the methodology that is used in each field of research. Parallelism is the area of research that parallels artists’ creations with functions of the brain; (Chatterjee, 2011b refers to this research that it “drapes art and aesthetics with neuroscience”). There are several scholars that promote the parallelist approach to neuroscience and art, such as Livingstone (1988), looking at the visual and perceptual processes involved in art experiences, or Ramachandran and Hirstein (1999), who introduced a “neurological theory of aesthetic experience”. Furthermore, the researcher who is most commonly associated with the parallelist approach is Zeki (1999); he is interested in studying the neural basis of art, and he believes that art is an “extension of the brain” as it employs the same functions and principles of the brain when art is created and when it is perceived. Knowledge creation is what Zeki assumes to be at the core of both the functioning of the brain and that of art. Zeki views the artist as a “naïve” neuroscientist who researches and uses his insights into visual processes and properties in creating an artwork, instead of studying it in a lab; however, his approach has also been criticized (e.g., Chatterjee, 2011a).

The other line of research that Chatterjee (2011a) differentiates is “experimental neuroaesthetics” which approaches the arts with testable hypotheses to be investigated using neuroscientific methods to further understand features of the aesthetic experience on the sensational, emotional (such as aesthetic emotion, pleasure, reward) and also on the semantic level. Traditionally, there have been fewer attempts to bridge the two disciplines from this angle and to approach art experience from a hypothesis-based neuroscientific perspective. However, this field has been rapidly growing in recent years and has produced promising insights into what art is, and what the crucial aspects of an art experience constitute. To date, several reviews and meta-analysis have been published, attempting to systemize and synthesize the insights that have been gained on the psychological and brain basis of interacting with art (e.g., Solso, 2001; Jacobsen et al., 2006; Di Dio and Gallese, 2009; Cela-Conde et al., 2011; Chatterjee, 2011a; Leder, 2013).

This approach corresponds partly to the one of our own research environment (e.g., Pöppel et al., 2013a). We use art as a potentially rich source of stimuli to obtain a better insight into cognitive mechanisms. We argue that perceptual stimuli from the arts like paintings, poetry or music allow a unique access to higher cognition complementing other experimental paradigms. We have given examples that demonstrate how useful such an alternative and complementary approach can be. For instance, how we perceive art depends on mental framing and sensory priming (Graupmann et al., 2013; Silveira et al., 2014a,b). Furthermore, a better understanding of personal and what is referred to as “the self” can be obtained by using artwork (Pöppel, 2010; Zaytseva et al., 2014; Zhou et al., 2014; Bao et al., 2016a). In addition, it could be demonstrated using surrealistic and naturalistic paintings that the brain distinguishes effortlessly between the physically possible and the impossible (Silveira et al., 2012). This observation may superficially relate to the fluency model (Reber et al., 2004) which is favored by many researchers in empirical aesthetics. However, we prefer to relate these results to the thought pattern of complementarity as it is shown that the brain distinguishes effortlessly between alternatives; presumably, for surrealistic art additional neural modules have to be switched on, which are active below threshold when viewing naturalistic pictures. This observation may relate to observations on aesthetic and moral judgments; it has been suggested that they share the same neural network, moral judgments coopting additional neural modules like the precuneus, the posterior cingulate cortex and the temporoparietal junction (Avram et al., 2013). It comes, however, as a surprise that this observation of sharing neural substrates should “substantiate the significance of symmetry and complexity for our judgment of beauty” as Jacobsen et al. (2006) suggest; it is difficult for us to follow this argument.

From our own research environment some additional observation might be of interest. With respect to the aesthetic appreciation of music it is suggested that personality factors are important predictors for perceptual processing (Park et al., 2013). Furthermore, individual imprinting is essential how we experience art (Park et al., 2014, 2015); long-term musical education makes us more sensitive to detect negative emotions like sadness in music or speech. With a new experimental paradigm using experiences stored in episodic memory (instead of pre-fabricated stimulus scenarios) it could be shown that retrospectively the beauty or ugliness of environments can be evaluated (Vedder et al., 2015). The visual field representation in the brain, in particular the hemispheric difference, has been shown to be reflected in pictures (Stoerig et al., 1983); pictures with a strong emotional appeal more often show their optical center on the left side corresponding to right hemisphere in neural processing. Different representations of visual perspectives in Eastern and Western art indicate substantial intercultural differences (Bao et al., 2016a); whereas the floating view in Chinese or Japanese landscapes creates an internal point of view (in German: “Ich-Nähe”), the central perspective in Western landscapes leads to an external point of view (“Ich-Ferne”). Within this context we argue that the central perspective does not mimic veridically the way of seeing as has been implied by some art scientists (e.g., Gombrich, 1982). The central perspective which follows geometrical rules does not account for size constancy, it does not appreciate distance effects as objects far away have less clear contours, and it misrepresents the visual field as its periphery extending up to 90 degrees visual angle along the horizontal meridian is contracted in pictures to a much smaller visual angle to be represented only in the perifoveal region. On a theoretical level it has been argued that the aesthetic principle can be used as a unifying concept of the different modes of knowledge (Pöppel and Bao, 2011). A unifying principle of our endeavor is to focus on anthropological universals and cultural or individual specifics in the arts, and how they allow a deeper understanding of cognitive mechanisms (Bao and Pöppel, 2012).

Taken together, neuroaesthetics in spite of all the criticism that has to be spelled out is an inspiring growing body of research that is exploring similarities between the functions of the brain and the ways artists work, as well as studying neurocognitive and neuroaffective correlates of aesthetic experiences. These approaches implicate a growing transdisciplinary interest neuroscientists and psychologists have in art and aesthetic appreciations. The following sections of our short summary are intended to illustrate the significance of neuroscience and its methods to gain insights into questions of art theory beyond neuroaesthetic parallelism. Most studies we mention focus on the neural underpinnings of the sensational, emotional, or semantic aspects of experiences with visual art.

### NeuroImaging Studies on Art and Non-art

One question that neuroimaging studies have been focusing on with regard to understanding art is very basic: what is art? How does the brain process art compared to non-art? What makes art different from other images or objects and what is the neural basis of viewing an original artwork compared to a copy or to non-art? A number of brain imaging studies have been conducted to investigate these questions to understand what makes an artwork different from images or objects that are not artistic creations. Most of these studies have compared images of original works to either manipulated version or non-art images or photographs. These studies yield insight for understanding what makes art special, what makes an experience with art special.

Di Dio et al. (2011) used fMRI as a method and compared the neural responses to images of classical male sculptures with non-artistic photographs of human bodies in a group of students that had no training in art or art history. When participants were asked to observe the original sculptures their neural responses yielded stronger activation in the antero-dorsal portion of the right insula compared to when they were observing the images of the human body. The authors argue that the original sculptures may possess features that are associated with aesthetic appreciation (golden ratio principle of canonical proportions) stressing the involvement of the insula, and they interpret this activation as a “hedonic signature of aesthetic experience”.

Similarly to this study, Lutz et al. (2013) conducted an fMRI study to investigate the differences in neural responses to images of body representations that are either visual artworks by artists such as Rubens, Kirchner, or non-artistic photographs. The authors report increased neural activation in the right parietal cortex and in bilateral extrastriate cortex in response to the artistic images. They interpret their findings as evidence that viewing an artwork involves distinct patterns of neural activation that may reflect processes such as visuo-spatial coding and also motor mapping.

Kirk et al. (2009) have approached the study of neural responses evoked by images that are labeled as art or non-art to understand cognitive “top-down” influences. The authors used fMRI to study how the neural responses of their participants differ between images that were either labeled as works of art from an art gallery or as generated by a computer. Participants observed the images in the scanner and the authors found stronger activation in the medial orbitofrontal cortex and prefrontal cortex in the art gallery context compared to the computer context. They suggest that their findings imply that the semantic context (information about validity as an artwork) modulates brain responses related to reward and thus crucially influences the aesthetic experiences.

Mizokami et al. (2014) investigated whether there are distinct neural underpinnings of viewing paintings, independently of their motif or of an assessment of beauty. The authors conducted an fMRI study and presented their participants’ images of 15 famous paintings and photographs of these 15 paintings. The results of the study revealed increased activation in bilateral cuneus and left lingual gyrus. The authors stress that this finding implies distinct neural mechanisms for aesthetic appreciation of representational paintings. A question that is closely related to the problem of “art or non-art” is directed to the artwork’s authenticity. A viewer’s experience and the respective neural response seems to be directly impacted by the declared authenticity of an artwork, whether it is a “real” work, or a fake or imitation. Huang et al. (2011) and Parker (2014) conducted an fMRI study that measured the brain activation patterns of art experts in response to several Rembrandt paintings that were either labeled to be authentic or to be a copy. In response to the authentic paintings, the authors found increased activation in orbitofrontal cortex and relate this response to the reward aspect of the viewing experience of a genuine artwork. In response to the artworks that were marked to be copies particularly the fronto-polar cortex and further brain areas showed increases in activation that may reflect a process of sequential thoughts.

Lacey et al. (2011) focused on a related question using fMRI: the authors investigated how the artistic status of an image influences the neural activation patterns when viewing the images. In the scanner, participants observed images of drawings and paintings from a variety of styles and of photographs that were classified as non-art images. The authors found that art images activated the ventral striatum, hypothalamus and orbitofrontal cortex, regions of the brain that are related to reward. The authors conclude that not the hedonic value but the artistic status of an image drives the visual art experience that the reward-related areas of the brain. Taken together, studies dealing with the neural correlates of art compared to non-art have stressed the reward value and emotional pleasure that an engaging with authentic and genuine art evokes; the studies mentioned above allude that brain areas related to specific cognitive and most importantly reward processes are involved when we engage with art, or with images that we take to be art.

### Themes from Art History and Art Theory

Any definition of art in art theory and philosophy is closely related to the identification of aspects that are crucial to a given understanding of art and features that ought to be focused on when interacting with or making statements about art. At present there have been few cross or inter-disciplinary approaches that have built on or have established theoretical connections between brain sciences and positions from art history or art theory. While John Onians (2008) “Neuroarthistory” is an example of blending art history with insights form brain sciences, it is mainly focused on the art historical context artists worked in and artworks were created in. However, a growing number of experimental studies from the field of neuroscience and psychology provide some insight into specific aspects that have been proposed to constitute art or are crucial for an understanding of art.

In the following section, we will point out aspects of some prominent areas of interest in art history and art theory and will elaborate ways neuroscientific investigations have provided valuable insights into certain brain mechanisms that might correlate with or underlie those ideas. A certain number of broad categories that may be useful in categorizing theories of art have been proposed and rely on broad terms such as presentation, expression, or form (e.g., Berleant, 1969; Osborne, 1970; Carroll, 1999).

#### Representation

Theories of art that assume that art at its essence is an attempt to depict reality and ought to reflect the real world can be described as representation theories of art. Idealists such as Plato for instance stress that art should replicate the ideal found in nature: art should transcend to a higher level, work as an ideal, and reflect non-humanly perfection in lacking expression and emotion. Winckelmann (1755) formulated the (possibly) first concise framework for art critique and history, investigating and analyzing ancient Greek art as the ideal model for art production. He proposed that beauty is in tension with expression and only few artworks achieve ideal beauty in that they are free of emotions or sensations, and he assumes that through “Nachahmung” (imitation) of nature idealization can be achieved. But such imitation of nature proves to be complex, as we need to define what kind of nature we are dealing with. Winckelmann relies on a medieval interpretation of Aristoteles’ reflections on a “poietic nature”, which led to two different ideas, i.e., “natura naturans” and “natura naturata” which is again an example of complementarity. While the Greek ideal of art as “naturing nature” in its essence and dynamic creativity was influential until romanticism, the “natured nature”, its domestication and objectification, is the guiding criterion of art since the enlightenment and the triumph of reason.

A variety of studies from neuroscience and psychology have investigated subjects related to the question of representation or idealization in art. Related to the idealist stance that art should reflect the true ideal of nature, the question of “an ideal” representation compared to an altered representation was investigated by Di Dio et al. (2007). The authors used images of renaissance and classical sculptures that represent idealistic properties of the human body and of modified versions of the same sculptures to investigate differences in brain responses of non-experts in art during their observation in an fMRI experiment. The authors report that observation of original sculptures led to activations in the lateral occipital areas, the precuneus and prefrontal areas and in particular in the right insula. The authors suggest that this activation is related to processing of objective beauty that an original artwork possesses, and that it is independent of a subjective aesthetic judgment.

Apart from the issue of an ideal representation of natural forms in art, another branch of research has been focusing on the questions of how the brain processes different artistic representations, and if there is a difference in neural processing between art works that depict a realistic image of a scene (“natura naturata”) and the natural scene itself. Vogt and Magnussen (2005) investigated hemispheric specialization for abstract and object based images. The results of their study indicated the two styles of images were processed with a different hemispheric advantage, and that these differences were modulated by artistic expertise.

Silveira et al. (2012) conducted an fMRI study and investigated the differences in brain responses to works of art that were either naturalistic or surrealistic (see above). Surrealistic and naturalistic images matched with regard to psychophysical parameters were presented to subjects with no background in the arts. The authors found increases of activation in the visual cortex and in the precuneus. The authors conclude that their findings may reflect decreased processing fluency for surrealist paintings, and increased self-referential processing in response to art works that portray realistic representations.

There are several studies that have compared brain responses to abstract vs. representational art. Cattaneo et al. (2014) used transcranial magnetic stimulation and were able to show that the left prefrontal cortex and the right posterior parietal cortex was differently engaged during viewing abstract and representational artworks and their activation depended on individual preferences. In another study, Cattaneo et al. (2015) used the same method to investigate the effects of brain stimulation during viewing abstract and representational painting, this time focusing on the role of the lateral occipital area which is involved in object recognition. The authors found that stimulation of the lateral occipital area during viewing of paintings reduced the aesthetic evaluation of representational paintings but not of abstract paintings. The authors conclude in stressing the importance of the lateral occipital areas in aesthetic appreciation of representational artworks due to the neural processes that are involved in object recognition.

Lengger et al. (2007) used slow cortical potentials (SCPs) to investigate differences in neural activation patterns in response to modern and contemporary representational art by artists such as Maria Lassnig, Peter Pongratz, Jean Michel Basquiat, and abstract artworks by artists such as Mark Rothko, Gerhard Richter, Yves Klein, and Jackson Pollock. Participants viewed the artworks and made ratings for understanding and aesthetic appeal. The authors found increased activation in response to representational paintings in the left frontal lobe and bilaterally in the temporal lobes and they suggest that these differences may be due to the lack of recognizable objects in abstract art that impedes the processing of abstract artworks.

Fairhall and Ishai (2008) studied the neural responses to representational, indeterminate and abstract art using fMRI. Indeterminate art represents a certain perceptual ambivalence (e.g., Muth and Carbon, 2016) in which objects are suggested but perceptually unseizable. The authors presented 12 subjects with 52 indeterminate paintings by Robert Pepperel, and 52 representational and abstract paintings, respectively. The authors found similar activation for all types of paintings in the visual cortex and the intra-parietal sulcus, probably reflecting processing of object form and challenging attentional demands. Representational artworks elicited stronger activation in the right fusiform gyrus, implying increased (top down) processing of familiar objects (including faces). Also, compared to indeterminate artworks, neural responses to representational paintings increased activation in the tempo-parietal junction, possibly reflecting processes of binding of form and spatial information. On the other hand, indeterminate artworks compared to the other types elicited the least activation in the right hippocampus, which may reflect impeded encoding of these artworks. The authors stress the involvement of a distributed cortical network that is involved in and modulated by the perception of different types of visual art. While neuroimaging studies may of course not be able to determine the artistic value of different types of representation in art, the studies reviewed above do support a stance that assumes fundamental differences in experiences with art influenced by ways of representation.

Although the studies referred to above (and many others not mentioned in this selected overview) which are employing modern experimental technologies appear to provide important new insights, some remarks on methodological problems are necessary. Transcranial magnetic stimulation is an interesting new technique (e.g., Cattaneo et al., 2014, 2015), and it is argued that it allows causal analyses of central information processing, but such conclusions have to be treated with caution. It might perhaps be concluded that the short-term interruption of neural activity in certain areas of the cortical mantle indicates a participation for a specific subjective experience, but because of the wide-spread blocking of neural activities which are not focussed on neural modules, it is difficult to argue that specific neural modules with their spatial characteristics are involved. Furthermore, from a methodological point far-reaching interpretations of results obtained with fMRI should also be treated with caution as has already been indicated above. It is unclear whether specific activation patterns represent an excitatory or an inhibitory component of neural activities; it could be simply neural participation of a large area which could have rather different meanings like central fatigue, or indeed perhaps on the psychological level more or less aesthetic appreciation or any other cognitive appraisal. Another problem with respect to investigate aesthetic appreciations are the typical stimulus presentations in scanning experiments which certainly do not reflect real life situations. Although such critical issues from the methodological perspective have to be raised, it should be stressed on the other hand that one cannot conclude that such studies are without value. In this field of research it is often the case that the results validate the methods. If one observes differences in neural activations the methods chosen have been proven to be useful otherwise one would not have obtained a result. However, whether the observed differences reflect a test of the hypotheses is a different issue. Indeed, the interpretations of results obtained in studies with imaging technologies are often rather far-reaching which include studies from our own research environment (e.g., Silveira et al., 2012). However, this does not allow the conclusion that the interpretations are wrong, but one has to be cautious with respect to their psychological implications.

#### Expression and Form

Another important aspect of art is its expressive potential. This is not directly related to the triggering of emotion in the perceiver but more specifically to the expression of the artist’s own emotions and the potential induction of the same in the beholder. Expression theories of art stress that the essence of art is “… as an expression of any kind of conscious experience – intellectual, emotional, or imaginative” (Khatchadourian, 1965), i.e., to effectively illuminate the inner states of the artist through a work of art. In fact, studies have shown, that viewers are capable to perceive and also largely agree on the emotions that are expressed in artworks. For instance, Blank et al. (1984) found a high consistency in identification of emotions in artworks. However, there are two ways the artist can express emotions through the artwork, i.e., through content and through form.

How do formal attributes influence neural responses to art? What different brain activation patterns are related to compositions of lines, shapes, colors, and other formal properties of an artwork? Representatives of formalism such as Bell (1914) assume that at the core, art is “significant form” and good art uses those formal elements to trigger an “aesthetic emotion” in sensitive observers. “Anything which is art is an instance of significant form; and anything which is not art has no such form” (Weitz, 1956). There has been recently increased attention on these topics in neuroscience (e.g., Chatterjee, 2003), but studies that have investigated emotional perception related to artworks, have mostly focused on the behavioral level. One study that stands out was conducted by Melcher and Bacci (2013). The authors pursued a truly interdisciplinary investigation and studied the perception of emotion in abstract art from a neuroscientific and art historian view. They report neural activation in the interior frontal gyrus in response to artworks that had been rated as highly emotional. The authors assume, that perception of emotion in artworks possibly recruits similar networks as those required for empathy processes. Furthermore, the authors developed a set of stimuli consisting of emotional abstract paintings that were pre-selected to express sadness and happiness and used these in an emotional priming study. The authors report that paintings were indeed successful as emotional primes. In a second study, the authors investigated the emotions subjects perceived in abstract paintings from visual cues such as lines, shapes, colors, depth and composition; they found that abstract artworks that were reported to express positive emotions included bright colors, complementary color contrasts and simple and regular shapes. The artworks that were rated to express negative emotions involved dark colors, and irregular shapes and forms. Furthermore, they were able to successfully train a computer algorithm to predict emotional perception ratings. The authors conclude, that there are indeed bottom-up features in abstract paintings that determine the emotions that viewers perceive in these artworks.

#### Embodiment and Simulated Motions

Related to the perception of emotion in artworks is the notion of embodiment, the empathizing with the artist through his creation. Freedberg and Gallese (2007) proposed that mirroring and simulating mechanisms in the brain are crucial aspects of emotional responses to art. The authors assume that viewers automatically and internally simulate the emotional expressions but also the movement represented or gestures implied in the artwork. They suggest that the perceived movements in the artwork evoke a feeling of bodily engagement in the observer that is reflected by brain activation in mirroring areas as well as emotional and motor related areas.

Battaglia et al. (2011) investigated the effects of movement represented in a painting on the motor system in a transcranial magnetic stimulation (TMS) study. The authors used Michelangelo’s *Expulsion From Paradise* as stimuli and compared the neural excitability in response to this painting, during imagery of the painting and while viewing a photographic reproduction of the painting. Also, they compared brain activity to images that showed the same body part (right hand) at rest and in a more emotional context. The results showed that the representation of movement in the original Michelangelo painting and during imagery led to increased cortical excitability and specifically during imagery of the painting, intracortical inhibition was reduced.

Umilta et al. (2012) also studied the relationship between movement and cortical motor activation. Other than the former study, they used abstract paintings that do not directly represent a bodily movement but merely imply an action. Using EEG the authors presented three high-resolution images of paintings by Lucio Fontana that show a varying number of cuts on a canvas and three visually modified versions of the paintings on a computer screen. The authors found that only the original artworks elicited activation in the motor cortex but not the modified versions, and the authors suggest that their findings support the proposition of internal simulation processes that are involved in the engagement with abstract visual art.

Sbriscia-Fioretti et al. (2013) further investigated this idea and focused on hand gestures. They conducted an EEG study with event related potentials and compared the cortical activation during passive observation of abstract paintings that included highly visible brushstrokes (visual indicators of hand movements) by the artist Franz Kline to modified versions of these paintings in which the “traces” of the artist movements were smoothed out. The results showed that the original paintings evoked stronger activation in the premotor and motor cortex as well as orbitofrontal and prefrontal areas. They relate these latter activations to reward related and cognitive processes and stress. In concluding, the authors stress the crucial roles that these activations together with the motor related activations play for the experience art and they interpret their findings as strong support for the proposition of embodied simulation of implied movements specifically in the processing of abstract art. Taken together, the studies referred to in this section lend support to the notion that interacting with a work of art may indeed involve the perception and identification of an (emotional) expression in a work of art, and the perception of this expression can be related to an embodied experience such as an internal simulation of the motions perceived in the artwork.

#### Affective Aesthetic Experiences

Closely related to focusing on the expressive force of an artwork is to approach art with an emphasis on the emotional response an artwork can evoke in the observer and his or her aesthetic experiences. The term “aesthetic” was first introduced by Baumgarten (1750/1758/2007), and is derived from the Greek “aisthesis” for “things perceived by the senses” instead of “things known by the mind”. An aesthetic response goes, however, beyond sensation and perception. Interacting with an artwork also has effects on the affective dimension and may lead to a strong emotional sensation. For instance, the romanticists proposed that the most important aspect of art is its capabilities to trigger strong affective responses in the perceiver. There is, however, another aspect of the romantic movement which supports our concept of complementarity as a generative principle which is “romantic irony” (Schlegel, 1798–1801/1988). Appreciating art has always the double aspect of being emotionally involved and having at the same time an ironic distance.

The essential function of art is seen in the expression of emotion instead of reflecting reality as it is. Burke (1757) introduced the concept of the sublime in aesthetics for distinguishing the beautiful and the sublime within their own categories, although we look at the beautiful and the sublime with the complementary thought pattern. The beautiful, according to Burke, is what is well-shaped, in proportion and aesthetically pleasing, whereas the sublime is a powerful force inspiring awe, and it has the potential to destroy us. Burke’s definition of the power of the sublime proved influential for the aesthetics of the Romantic era. For Burke (1757) in turn, beauty is a universal concept and lies in the perceiver as he interacts with it; beauty is regarded to be “ohne Interesse” (without interest). There is a large and growing body of research using neuroimaging techniques to study aspects of affective aesthetic experiences “with interest”, i.e., the strong emotional responses to artworks. Vessel et al. (2013) summarized aesthetic experience as a research topic as follows: an “aesthetic experience involves more than preference, encompassing a variety of emotional responses ranging from beauty to awe, sublimity, and a variety of other (often knowledge-based) emotions”.

It has been proposed that the experience of art is associated with activity in a network of brain areas and that the pleasure and positive feelings of an aesthetic experience rely on the reward circuit, including cortical and subcortical regions such as the orbitofrontal cortex and the ventromedial prefrontal cortex (that are strongly related to reward processing and have consistently been related to the assessment of beauty and aesthetic appeal), the anterior cingulate cortex (that has been associated with preferences and liking in different artistic domains and its activation has been assumed to reflect the state of a person’s subjective feeling), the insular cortex (that has been consistently associated with self-referential processes and the experience of emotions), and the nucleus accumbens (which is said to be involved in the generation of positive affective experiences and is assumed to play a role in the detection of the emotional value of an artwork) (e.g., Cela-Conde et al., 2004; Kawabata and Zeki, 2004; Vartanian and Goel, 2004; Jacobsen et al., 2006; Cinzia and Vittorio, 2009; Ishizu and Zeki, 2011; Nadal, 2013). Corroborating this view, Brown et al. (2011) conducted a meta-analysis of 93 neuroimaging studies and concluded that aesthetic experience crucially relies on an appraisal process of the affective value of a work of art (or other aesthetic stimuli) that reflects intero- and exteroceptive processing, and at its heart involves the orbitofrontal cortex, the anterior insula, the rostral cigulate cortex and the ventral basal ganglia. In line with this model, Di Dio (2012) specified that particularly the activation of the anterodorsal sector of the right insular might constitute the core of the aesthetic experience reflecting the specific hedonic response toward an aesthetically pleasing work of art.

Recently, Vessel et al. (2012, 2013) have proposed that certain areas that are part of the default mode network (DMN) are crucially involved in aesthetic experience, specifically in highly intense emotional responses. In an fMRI study the authors found that while activity in other regions such as the striatum varied linearly with participants ratings of their subjective aesthetic response (how strongly they were moved by the artwork), specifically activity in the DMN increased stepwise only in response to artworks that were rated highest. The authors conclude that these activations reflect that aesthetic experiences with highly moving artworks contain self-referential processes that are strongly marked by subjective relevance of the works.

Cela-Conde et al. (2013) also investigated the involvement of the DMN in aesthetic experience but used MEG (magnetoencephalography) to study its temporal component. In three temporal windows and between two conditions, the authors found two different networks to be involved in aesthetic appreciation and thus propose a “twofold model of aesthetic appreciation” that involves an “initial” network mainly connecting occipital regions with links to the orbitofrontal cortex and a “delayed” network that includes parts of the DMN. The authors argue that this activation in stimulus dependent aesthetic processing might be related to internal mind wandering.

Silvia (2005) notes that apart from pleasure, an aesthetic experience with an artwork may also evoke other emotional responses in the perceiver such as enjoyment, interest, and distinct emotions such as sadness or joy. In this fashion, Silvia and Brown (2007) and Silvia (2009) also investigated other emotional responses during an aesthetic experience such as anger, confusion, or disgust. While there is a large body of neuroimaging studies on the perception and experience of emotions such as happiness, fear, sadness, surprise, amazement in the context of music (e.g., Juslin and Västfjäll, 2008; Zentner et al., 2008; Eerola and Vuoskoski, 2013; Koelsch, 2014; Park et al., 2014, 2015), there are as of now apparently (to the best of our knowledge) no such studies that focus on visual art.

## Complementarity in Interactions Between Artists and Scientists, and Future Challenges

In this summary above we focused on psychological and neural aspects of visual art. In so doing we neglected relationships between the different modes of art that have to be considered as characteristic for visual art, music, poetry or dance. One such fundamental link is their embeddedness in the temporal domain. Although visual art is mostly considered as “spatial art”, and music and poetry are “temporal arts”, this distinction is rather superficial, and even wrong. Sensory processing is always multimodal which is dictated by the neuro-anatomical connectivity between different brain areas, and time windows play an important role in all sensory domains as platforms for sensation and perception (Bao et al., 2015) proving again our concept of complementarity. One such time window has a duration of approximately 3 s as indicated above which can be observed in perception, movement control, sensorimotor synchronization, working memory, or spontaneous speech (Pöppel, 2009a; Pöppel and Bao, 2014), and this time window is also basic for the appreciation of music (Pöppel, 1989b) or poetry (Turner and Pöppel, 1988). It has been suggested (Pöppel and von Stosch, 2014) that this time window provides also a temporal frame for the appreciation of visual art like in cubism or surrealism. Pictures for instance of Pablo Picasso, Paul Klee, or Lyonel Feininger using ambiguous geometric figures like the Necker cube create in the viewer temporal instabilities because of spontaneous reversals; thus, one picture becomes in fact many pictures in the mind of the viewer in an unpredictable way.

This underlying connection between spatial and temporal processing in the arts appears to be an “unasked question” (Pöppel et al., 2013b), as indeed several other unasked questions can be identified in neurosciences or psychology. With respect to temporal perception continuous processing is usually assumed implicitly, in spite of the evidence of discrete processing as demonstrated by time windows of different durations (Bao et al., 2015). Another unasked question refers to the implicit assumption of homogeneity of space, although it has been demonstrated that visual space with respect to attentional control is not at all homogeneous (Bao and Pöppel, 2007); in fact, as one refers to “time windows” in sensory processing in the temporal domain, one has to refer to “space windows” in the spatial domain; within the perifoveal region sensory information is processed differently compared to the periphery of the visual field, and this “eccentricity effect” has been documented by a number of studies (e.g., Bao et al., 2013a,b). As has been indicated above with respect to the “central perspective” in Western art, the space windows are also important for aesthetic appreciations; in typical pictures of Western landscapes the different space windows are loosing their perceptual saliency which is characteristic for human vision. We would like to submit that the intrinsic relationship between time windows and space windows in their complementarity opens new opportunities for research in visual art.

Our analysis and description of results certainly suffers from several deficiencies, but one deficiency is usually not addressed by others which is the strong language bias. This text is written in English and most references cited have been written in English with a few exceptions of original contributions in Chinese, German, Greek, Latin, Russian, or Spanish. It is usually implied, again reflecting an unasked question, that languages are neutral with respect to the content to be expressed. If one has to write in a foreign language one realizes that the language is not neutral at all with respect to what one wants to say; certain terms are extremely difficult or impossible to translate into another language like for instance the temporal marker “present” which in its Chinese and German “translations” evoke rather different connotations (Zhou et al., 2014). Does the English word “beauty” actually define subjectively the same frame of reference as “Schönheit” in German? How does one translate the German “ästhetische Empfindungen” (Liessmann, 2009) into English? “Aesthetic sensibilities” as one might consider is not quite convincing; the German term has a more active, the English term a more passive flavor. Liessmann defines some important areas for empirical research when he refers to aesthetic appreciations not only within the context of “beauty”, but also boredom; but then again major language problems arise when he refers to “Anmut” (suggestions for English translations are “charm, grace, agreeableness,.”.), or “Rührung” (the suggested translation “emotion” misses completely the point). The famous dissertation of Worringer (1908/2007) has the title in German “Abstraktion und Einfühlung”, and the German “Einfühlung” is translated as “empathy” which for somebody with German as a mother tongue also misses the point. We raise this critical issue here only with respect to two languages; it applies of course to all languages which have to be mapped for communication purposes onto the dominant language in use.

But this is only one aspect of the language bias, i.e., being caught in different frames of connotations because of one’s mother tongue. The other bias is equally serious; we as authors, (and this applies to anybody working in this field), have neglected publications which have been written in languages we could not read; who would claim that there is not important information available on our topic written in French, Italian, Russian, Turkish, Japanese, or else, and which is not available in translations we can read, in spite of the deficiencies of translated texts? There is no hope that these language biases can be overcome.

But is there indeed no hope? It will not be found in rational conjectures within the conceptual jail of one language. In spite of all the critical points we have raised there is at least one trajectory that we pursue ourselves, and which we share presumably with many researchers in empirical aesthetics, and this is the complementary interactions between artists and scientists. On this personal level, complementarity as a creative or generative principle gets practical meaning. On this level of individual interactions scientists learn from artists, and artists learn from scientists; scientists are open to what artists are doing, and artists are curious about basic research. As it happens, the product of such creative co-operations is not necessarily represented in scientific publications, but rather in contributions of catalogs of the artists. In our own research environment the principle of this kind of complementarity came to life in poetry (Turner and Pöppel, 1988), in music with the musician Herbert von Karajan (Pöppel, 1988), in visual art with the Russian/German artist Igor Sacharow-Ross (Pöppel and Ruhnau, 2006; Pöppel, 2009b), or in installations with the Icelandic/Danish artist Olafur Eliasson (Pöppel, 2011). At present we are looking for neural correlates of aesthetic appreciations in the work of the Chinese artist LaoZhu who represents the “third abstraction” (Bao et al., 2017). According to some art historians the work of Malevich, Kandinsky, or Mondrian represents the first abstraction, the cubism of Picasso and the abstract expressionism of Pollock stands for the second abstraction. Whereas the first and the second abstraction focus on shapes and colors with meaning or being meaningless, the third abstraction emphasizes the internal point of view, and it is focused on emotional appreciations which can already be seen in the work of Malevich. In the third abstraction pictures create a feeling of belongingness, and, thus, they stabilize personal and cultural identity. It is on this personal level of complementarity where we get personal satisfaction and reward, and where new ideas are born in an unpredictable way which then can be investigated within an empirical context.

## Author Contributions

YB and EP developed the theoretical concept. AvS contributed viewpoints from art history and art science. MP summarized the empirical research on neuroaesthetics. YB and EP wrote the final manuscript.

## Conflict of Interest Statement

The authors declare that the research was conducted in the absence of any commercial or financial relationships that could be construed as a potential conflict of interest.
